# Awareness and perceptions of nicotine replacement therapy among adults in Saudi Arabia: A cross-sectional study

**DOI:** 10.18332/tid/221483

**Published:** 2026-08-01

**Authors:** Fatma I. Almaghlouth, Hajed M. Al-Otaibi, Malik A. Althobiani, Abdulaziz Albalawi, Fanar Malibari, Nawaf Alqarni

**Affiliations:** 1 Department of Respiratory Therapy, Faculty of Medical Rehabilitation Sciences, King Abdulaziz University, Jeddah, Saudi Arabia

**Keywords:** nicotine replacement therapy, smoking cessation, awareness, nicotine dependence, Saudi Arabia

## Abstract

**INTRODUCTION:**

Nicotine replacement therapies (NRTs) are established evidence-based interventions for smoking cessation. In Saudi Arabia, where tobacco use remains a significant public health concern, understanding public awareness, perceptions, and motivations related to NRT use is essential for designing effective cessation strategies. This study aimed to assess awareness, perceptions, motivation to quit smoking, and self-reported nicotine dependence related to NRT use among adults in Saudi Arabia.

**METHODS:**

Between February and May 2025, a cross-sectional online survey was distributed via social media platforms to adults aged ≥18 years across Saudi Arabia. The survey comprised four domains: demographics, NRT awareness/perception, the Questionnaire on Motivation to Quit (Q-MAT; 4 scored items, range 0–20), and the Nicotine Dependence Syndrome Scale (NDSS; 16 items, 5-point Likert scale). The Q-MAT and NDSS were administered only to current smokers. Internal consistency was assessed using Cronbach’s alpha. Chi-squared tests examined associations between demographic variables and NRT awareness. Binary logistic regression identified predictors of NRT awareness. Independent samples t-tests compared NDSS scores by motivation level.

**RESULTS:**

Among 425 eligible participants, 72.5% were male, and the largest proportion were aged 25–34 years (43.3%). Overall, 89.1% of the participants were aware of NRTs. However, 49.3% were unsure about the most effective NRT type, and 37.3% were uncertain about its side effects. The Q-MAT (Cronbach’s α=0.729) yielded a mean motivation score of 12.3 ± 5.8 (out of 20), with 44.4% classified as highly motivated. The NDSS (α=0.876) revealed moderate-to-high dependence (overall mean 2.94 ± 0.78), with the Drive subscale scoring highest (3.25 ± 1.09). Logistic regression showed male gender was a significant predictor of NRT awareness (OR=1.98; 95% CI: 1.01–3.89; p=0.048). Two-thirds of participants (66.0%) supported NRT integration into academic curricula.

**CONCLUSIONS:**

While general NRT awareness was high among our sample of Saudi adults, significant knowledge gaps persist regarding its effectiveness and safety. High motivation to quit combined with moderate-to-high nicotine dependence underscores the importance of this area for future investigation. Longitudinal and interventional studies are needed to determine whether targeted education, improved access to cessation support, and integration of smoking cessation content into healthcare training programs can effectively address these gaps.

## INTRODUCTION

Tobacco use remains one of the leading preventable causes of morbidity and mortality worldwide. According to the World Health Organization (WHO), tobacco use accounts for over eight million deaths annually^1^. In Saudi Arabia, despite ongoing governmental efforts to curb tobacco consumption through regulatory frameworks and public health campaigns^2^, smoking prevalence continues to represent a major health challenge. Recent estimates suggest that approximately 12–21% of the Saudi adult population engages in some form of tobacco use, with wide variation across regions, age groups, and socioeconomic groups^3^.

Nicotine replacement therapies (NRTs) have been recognized globally as first-line pharmacological interventions for smoking cessation. These products, including nicotine patches, gum, nasal sprays, inhalers, and lozenges, deliver controlled doses of nicotine to alleviate withdrawal symptoms and reduce cravings associated with tobacco abstinence^4,5^. Systematic reviews have reported that NRTs increase the likelihood of successful quit attempts by 50–70% compared to placebo or no pharmacotherapy^6,7^. More recently, nicotine pouches have entered the Saudi market as consumer products and promoted by manufacturers as a form of harm reduction^8,9^; however, these are not classified as pharmacological NRT, and their role within formal cessation programs has not yet been established.

In the Saudi context, several factors affect NRT adoption and effective use. Cultural norms, gender-specific behaviors, and socioeconomic factors influence smoking patterns and cessation behaviors. Al-Nimr et al.^10^ identified that cultural expectations and social environments significantly influence smoking initiation and cessation among Saudi women. Tobaiqy et al.^3^ noted gaps in awareness and utilization of evidence-based cessation resources, highlighting the need for culturally sensitive health education strategies.

Evidence suggests that public awareness and understanding of NRT remain limited. Monshi et al.^11^ analyzed data from the 2019 Global Adult Tobacco Survey and reported that only 60% of Saudi tobacco users were aware of smoking cessation clinics, underscoring gaps in knowledge about available cessation resources. Similarly, Almehmadi et al.^12^ reported that only 60.6% of medical students at a Saudi university were aware of NRT, with senior students showing significantly higher awareness than juniors. Furthermore, Alzahrani et al.^13^ found that community pharmacists frequently encountered customers lacking understanding of NRT mechanisms.

Despite the growing availability of NRT products in Saudi Arabia, evidence regarding how these products are perceived, understood, and utilized remains limited. Moreover, understanding public motivation to quit, misconceptions about NRT efficacy and safety, the severity of nicotine dependence, and attitudes toward NRT use in vulnerable groups need to be determined. This study, therefore, aimed to assess the awareness, perceptions, motivation to quit smoking, and self-reported nicotine dependence related to NRT use among adults in Saudi Arabia.

## METHODS

### Study design

This study employed a cross-sectional descriptive design using an online self-administered survey to assess awareness, perception, motivation to quit smoking, and nicotine dependence patterns related to NRTs among adults in Saudi Arabia.

### Participants and sampling

Adults, Saudi residents aged ≥18 years, were invited to participate through a survey distributed across multiple regions of Saudi Arabia using a convenience sampling strategy. Data were collected from February to May 2025. The survey was distributed online via social media platforms, including Telegram, WhatsApp, and Twitter (X), and was also administered in person using tablet devices (iPads) by research assistants who approached participants at the university campus. Adolescents younger than 18 years, those who were unable to sign the consent form, and incomplete responses, were excluded.

### Data collection instrument

The survey comprised four domains. The first domain collected demographic information including age (18–24, 25–34, 35–44, 45–54, ≥55 years), gender (male, female, prefer not to say), marital status, education level (high school or lower, diploma, Bachelor’s, Master’s, PhD), employment status (employed, unemployed, student, retired), monthly income in Saudi Riyals (<5000, 5000–10000, 10000–20000, >20000), chronic disease status (yes, no), and smoking behavior. The second domain assessed NRT awareness and perception through 14 items covering knowledge of NRT types, perceived effectiveness, awareness of potential side effects, application methods, and opinions about NRT use in academic and clinical contexts. Demographic variables (gender, age group, and education level) were examined as potential predictors of NRT awareness; no additional variables were treated as confounders in the present analysis, as only bivariate associations and unadjusted logistic regression models were employed.

The third domain evaluated motivation to quit smoking among current smokers only, using items adapted from the Questionnaire on Motivation to Quit (Q-MAT), a validated instrument originally developed by Aubin et al.^14^. The Q-MAT items were translated into Arabic by the bilingual research team to ensure linguistic clarity and accuracy. Four scored items were included: (Q1) six-month quit intention, scored 0–8; (Q3) current desire to quit, scored 0–3; (Q4) four-week quit intention, scored 0–6; and (Q6) dissatisfaction with smoking, scored 0–3. Total Q-MAT scores range from 0 to 20, with higher scores indicating greater motivation. Scores were categorized as low (0–6), moderate (7–13), and high (14–20).

The fourth domain assessed nicotine dependence among current smokers only, through 16 items adapted from the Nicotine Dependence Syndrome Scale (NDSS)^15^. Items were rated on a five-point Likert scale from 1 ‘Not at all true’ to 5 ‘Extremely true’. Five subscales were computed: Drive/Craving (5 items), Priority (2 items), Tolerance (2 items), Continuity (3 items), and Stereotypy (4 items). Mean subscale and total NDSS scores were calculated.

### Statistical analysis

Data were exported from Survey Monkey and analyzed using IBM SPSS Statistics (Version 29.0, IBM Corp., Armonk, NY, USA). Descriptive statistics included frequencies, percentages, means, standard deviations, and medians. Internal consistency reliability was assessed using Cronbach’s alpha (≥0.70 = acceptable). Chi-squared (χ^2^) tests examined bivariate associations between demographic variables and NRT awareness. Binary logistic regression was used to identify predictors of NRT awareness; each predictor (gender, age group, and education level) was entered in a separate univariable model, and crude odds ratios (OR) with 95% confidence intervals (CI) are reported. No multivariable adjustment for additional confounders was performed. Independent samples t-tests compared the mean NDSS scores between motivation groups. Statistical significance was set at p<0.05. Smoking status was inferred from completion of the smoker-specific Q-MAT and NDSS sections, as the explicit smoking status question (Q9) was affected by a skip-logic routing error in SurveyMonkey (only 3 of 467 respondents answered this item). Participants who completed the Q-MAT section were classified as current smokers; those who did not were classified as non-smokers.

### Ethical considerations

Participation was voluntary, and electronic informed consent was obtained prior to survey access. The study received ethical approval from the Research Ethics Committee (REC), Unit of Biomedical Ethics, Faculty of Medicine, King Abdulaziz University Hospital (KAUH), King Abdulaziz University, Jeddah, Saudi Arabia (Reference No. 17-25, NCBE Registration No. HA-02-J-008, approved 14 January 2025). All data were anonymized and securely stored.

## RESULTS

### Participant flow and demographic characteristics

A total of 467 individuals responded to the survey. Of these, 457 (97.8%) provided informed consent. After applying the inclusion criteria, 32 respondents were excluded; 9 were minors (aged <18 years), and 23 submitted incomplete responses, yielding 425 eligible participants for analysis. Therefore, the overall survey completion rate was 90.9% (425 of 467 respondents). Out of the analyzed sample, 359 (84.5%) completed domain 2 (NRT awareness and perception), representing the analytic sample for awareness-related outcomes. Among those routed to the smoker-specific sections, 219 completed the Q-MAT (Domain 3), and 175 completed the NDSS (Domain 4) with complete data on all items. Minor variations in item-level sample sizes (e.g. 219–223 for individual Q-MAT items, 175–183 for NDSS items) are noted in the respective tables.

Most participants were male (n=308; 72.5%), and the largest age group was 25–34 years (n=184; 43.3%). Most held a Bachelor’s degree (n=185; 43.5%) or a Master’s degree (n=103; 24.24%). Employment was reported by 289 participants (68%), and 84 (19.7%) were students. Based on completion of smoker-specific survey sections (Q-MAT and NDSS), 220 participants (51.8%) were classified as current smokers, while 205 (48.2%) were classified as non-smokers. A small proportion of participants (13.8%) reported chronic diseases. The complete demographic profile is presented in Table 1.

**Table 1:** Demographic characteristics of adult participants in a cross-sectional online survey on nicotine replacement therapy awareness, Saudi Arabia, February–May 2025 (N=425)

*Characteristics*	*Category*	*n*	*%*
**Gender**	Male	308	72.5
	Female	111	26.12
	Prefer not to say	6	1.41
**Age** (years)	18–24	103	24.24
	25–34	184	43.29
	35–44	103	24.24
	45–54	18	4.24
	≥55	8	1.88
**Education level**	High school or lower	58	13.65
	Diploma	39	9.18
	Bachelor’s	185	43.53
	Master’s	103	24.24
	PhD	37	8.71
**Employment**	Employed	289	68.00
	Unemployed	48	11.29
	Student	84	19.76
	Retired	4	0.94
**Monthly income** (SAR)	<5000	134	31.53
	5000–10000	84	19.76
	10000–20000	132	31.06
	>20000	75	17.65
**Chronic disease**	Yes	59	13.88
	No	366	86.12
24.24	Smokers (completed Q-MAT)	220	51.76
	Non-smokers	205	48.24

† Smoking status was inferred from completion of smoker-specific survey sections (Q-MAT and NDSS), as the explicit smoking status question (Q9) was affected by a skip-logic routing error (only 3 responses recorded). Participants who completed the Q-MAT section (n=219) were classified as smokers; those who did not were classified as non-smokers. Minor item-level variation (219–223) is attributable to item-level non-response on individual questions. Q-MAT: Questionnaire on Motivation to Quit. NDSS: Nicotine Dependence Syndrome Scale. SAR: 1000 Saudi Riyals about US$270.

### Awareness and perception of NRT

Among the 359 participants who responded to Domain 2, 320 (89.1%) reported awareness of NRTs. When asked about available forms of NRT, 156 participants (43.4%) identified patches and gum, 131 (36.49%) correctly identified all forms, and 60 (16.7%) reported no knowledge. Regarding perceived effectiveness, 179 participants (49.8%) believed that NRTs were effective, while 177 participants (49.3%) could not determine which NRT type was most effective. A total of 165 participants (45.9%) believed that NRTs could cause adverse effects, while 134 participants (37.3%) were unsure. Most participants (n=255; 71.0%) endorsed gradual dose tapering. Two-thirds of respondents (n=237; 66.0%) supported integrating NRT into academic curricula, and 195 participants (54.3%) were unsure about NRT safety during pregnancy. Table 2 presents awareness and perception among participants.

**Table 2:** Awareness and perception of nicotine replacement therapy among adult participants in a cross-sectional online survey, Saudi Arabia, February–May 2025 (N=359)

*Item*	*Response*	*n*	*%*
**Heard of NRT**	Yes	320	89.14
	No	39	10.86
**NRT perceived as effective**	Yes	179	49.86
	No	92	25.63
	Don’t know	88	24.51
**Most effective NRT type**	Gum	95	26.46
	Patches	77	21.45
	Don’t know	177	49.30
**NRT may cause side effects**	Yes	165	45.96
	Don’t know	134	37.33
**Gradual dose tapering necessary**	Yes	255	71.03
**NRT + counselling higher success**	Both together	170	47.35
**NRT in academic curricula**	Yes	237	66.02
**NRT safe during pregnancy**	Don’t know	195	54.32

NRT: nicotine replacement therapy.

### Associations between participants’ demographics and NRT awareness

Chi-squared tests revealed no statistically significant association between NRT awareness and gender (χ^2^=2.41; p=0.121), age group (χ^2^=2.20; p=0.138), or education level (χ^2^=0.82; p=0.365). Table 3 presents the association between participants’ demographics and NRT awareness.

**Table 3:** Association between participants’ demographics and nicotine replacement therapy awareness in a cross-sectional online survey, Saudi Arabia, February–May 2025 (N=346)

*Variable*	*Aware n (%)*	*Not Aware n (%)*	*χ²*	*df*	*p*
**Gender**			2.406	1	0.121
Male	236 (91.8)	21 (8.2)			
Female	76 (85.4)	13 (14.6)			
**Age** (years)			2.197	1	0.138
18–34	224 (91.8)	20 (8.2)			
≥35	92 (86.0)	15 (14.0)			
**Education level**			0.822	1	0.365
Lower than Bachelor’s	66 (86.8)	10 (13.2)			
Bachelor’s and higher	248 (91.2)	24 (8.8)			

Binary logistic regression identified male gender as being associated with higher odds of NRT awareness (OR=1.98; 95% CI: 1.01–3.89; p=0.048), indicating males had approximately twice the odds of being aware of NRTs compared to females. Age ≥35 years (OR=0.56; 95% CI: 0.27–1.17; p=0.123) and Bachelor’s degree and higher levels (OR=1.78; 95% CI: 0.82–3.87; p=0.143) were not statistically significant predictors. Table 4 presents the predictors of NRT awareness by gender, age group, and education level.

**Table 4:** Binary logistic regression: predictors of nicotine replacement therapy awareness in a cross-sectional online survey, Saudi Arabia, February–May 2025 (N=346)

*Predictor*	*OR*	*95% CI*	*SE*	*p*
**Sex**				
Female (ref.)	1			
Male	1.977	1.006–3.887	0.345	0.048*
**Age (years)**				
18–34 years (ref.)	1			
Age ≥35 years	0.563	0.271–1.169	0.373	0.123
**Education level**				
Lower than Bachelor’s (ref.)	1			
Bachelor’s and higher	1.784	0.822–3.872	0.394	0.143

SE: standard error. NRT: nicotine replacement therapy.

* p<0.05.

### Motivation to quit smoking

The Q-MAT was completed by 220 active smokers with acceptable internal consistency (Cronbach’s α=0.729, n=214 with complete data on all 4 scored items). The mean total score was 12.36 ± 5.89 (median=12.0, range 0–20). When categorized as low, moderate, or high motivation, 40 participants (18.7%) had low motivation, 79 (36.9%) moderate, and 95 (44.4%) high. Table 5 presents Q-MAT composite scores and motivation categories.

**Table 5:** Questionnaire on Motivation to Quit (Q-MAT) composite scores and motivation categories among current smokers in a cross-sectional online survey, Saudi Arabia, February–May 2025 (N=214)

*Measure*	*Value*		
Mean ± SD	12.36 ± 5.89		
Median (range)	12.0 (0–20)		
Cronbach’s α	0.729		
***Category***	***Score range***	***n***	***%***
Low motivation	0–6	40	18.7
Moderate motivation	7–13	79	36.9
High motivation	14–20	95	44.4

Chi-squared analysis showed no significant association between motivation level and age group (χ^2^=1.28; p=0.258). The full item-level response distribution for all Q-MAT items, including the three descriptive (non-scored) items, is presented in Supplementary file Table 1.

### Nicotine Dependence Syndrome Scale

**Figure 1:**
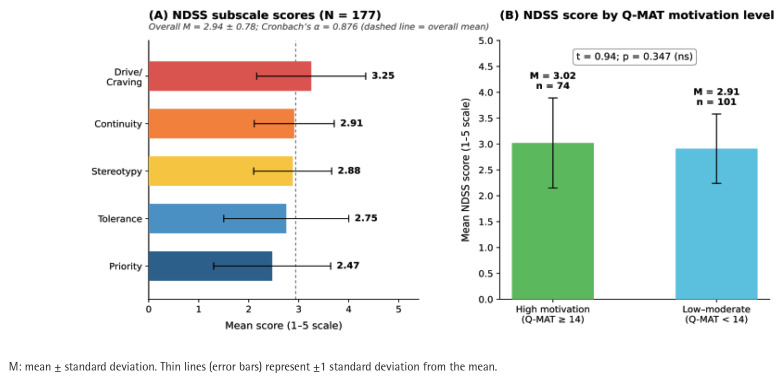
Nicotine Dependence Syndrome Scale (NDSS) results among current smokers in a cross-sectional online survey, Saudi Arabia, February–May 2025: (A) subscale mean scores with standard deviations; (B) comparison of NDSS scores by Questionnaire on Motivation to Quit (Q-MAT) motivation level

The 16-item NDSS demonstrated good internal consistency (Cronbach’s α=0.876, n=175 with complete data on all items). The overall mean NDSS score was 2.94 ± 0.78 on a 1–5 scale. Among subscales, Drive/Craving scored highest (3.25 ± 1.09), followed by Continuity (2.91 ± 0.80), Stereotypy (2.88 ± 0.78), Tolerance (2.75 ± 1.25), and Priority (2.47 ± 1.17). Figure 1 illustrates nicotine dependence syndrome scale results.

At the item level, 56.1% rated strong nicotine need after abstinence as ‘very’ or ‘extremely true’, and 49.4% reported emotional/situational influence at similar levels. Supplementary file Table 2 presents the NDSS item-level response distribution.

An independent t-test comparing NDSS scores between high-motivation (mean=3.02 ± 0.87; n=74) and low-to-moderate motivation groups (mean=2.91 ± 0.67; n=101) revealed no statistically significant difference (t=0.94; p=0.347).

### NRT in academic and sensitive contexts

**Figure 2:**
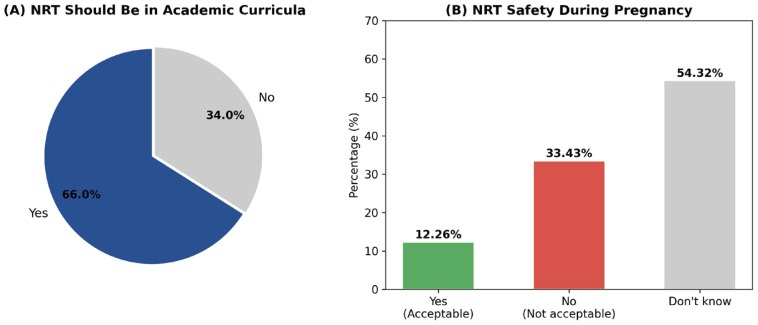
Opinions on nicotine replacement therapy (NRT) in special contexts among adult participants in a cross-sectional online survey, Saudi Arabia, February–May 2025 (N=359): (A) support for NRT in academic curricula, (B) perceptions of NRT safety during pregnancy

A total of 237 participants (66.0%) supported integrating NRT education into academic curricula, while 122 participants (33.9%) disagreed. Regarding NRT use during pregnancy, only 44 of the participants (12.2%) believed it was acceptable, while 120 of them (33.4%) considered it unacceptable, and 195 (54.3%) were unsure. Figure 2 illustrates the opinions of participants about incorporating NRT within academic curricula and safety during pregnancy.

## DISCUSSION

The present study assessed NRT awareness, perceptions, motivation to quit, and nicotine dependence among a sample of adults in Saudi Arabia using validated instruments with demonstrated internal consistency. The key findings reveal that while general NRT awareness was high within our convenience sample, detailed knowledge regarding specific product types, proper usage, and safety profiles remained limited. Male gender was the sole significant predictor of NRT awareness. The Q-MAT indicated that a substantial proportion of smokers were highly motivated to quit, while the NDSS revealed a craving-dominant dependence profile. No significant difference in dependence scores was observed between high- and low-motivation groups.

The high awareness rate observed in this study exceeds rates reported in earlier Saudi studies, possibly related to the sampling strategy, and is consistent with international evidence showing that smokers’ perceptions of NRT harmfulness influence their use as cessation aids^2,16,17^. Alrowais et al.^18^ found that clients attending smoking cessation clinics in Riyadh had limited knowledge about available NRT services, which might contribute to low utilization rates. However, awareness alone does not usually translate into effective utilization^19^. In the present data, nearly half of the participants were unable to identify the most effective NRT type, and approximately one-third correctly identified all available forms. This disparity has also been documented by Tobaiqy et al.^3^ who noted significant inconsistencies in awareness and understanding of smoking cessation methods across the Saudi population.

The logistic regression analysis identified male gender as a significant predictor of NRT awareness, possibly reflecting differential exposure to NRT marketing or healthcare encounters. Although education level and age were not statistically significant in the model, observed trends should be explored in larger studies with broader variables in the analysis.

Uncertainty surrounding NRT safety represents a modifiable barrier to adoption. More than one-third of respondents were entirely unsure about NRT side effects, and nearly half believed that NRTs could cause adverse effects. Evidence shows that NRTs carry fewer health risks than continued tobacco use^4^, and corrective messaging about nicotine can positively shift harm perceptions^17,20^. Alzahrani et al.^13^ found similar confusion among pharmacy customers, suggesting that safety misconceptions remain a common barrier to NRT adoption.

The Q-MAT analysis revealed moderate-to-high motivation scores with acceptable internal consistency, and nearly half of smokers were classified as highly motivated. These rates are comparable to structured cessation programs^21^. The finding that the majority of the group had attempted quitting multiple times indicates personal experience of smoking cessation difficulty and likely receptivity to pharmacological support.

The NDSS demonstrated good internal consistency and revealed a craving-dominant dependence profile, with the Drive subscale scoring highest among all subscales. This pattern may be relevant when considering pharmacological cessation support for this population. The comparatively lower Priority subscale may reflect Saudi Arabia’s recent smoke-free policies. Moreover, the present investigations showed no statistically significant NDSS difference between high-motivation and low-motivation groups, indicating that the relationship between motivation levels and severity of dependence remains unclear. This also suggests that motivation alone might be insufficient for smoking abstinence, and pharmacological support could make a meaningful difference in the smoking cessation process^22^.

The present data show that the majority of respondents supported incorporating NRT in academic curricula, which is consistent with the work of others^12^. Moreover, uncertainty about the safety of NRT use during pregnancy is consistent with findings from other countries, where evidence suggests that medically supervised NRT may be safer than continued smoking during pregnancy^23^.

### Strengths and limitations

Key strengths include the use of validated instruments (Q-MAT and NDSS) with demonstrated reliability, composite scoring with categorical classification, NDSS subscale profiling, and the incorporation of inferential statistics beyond purely descriptive reporting. However, limitations include the cross-sectional design, which precludes causal inference; convenience sampling (which may lead to sampling bias), which limits generalizability; and a SurveyMonkey skip-logic routing error that prevented direct recording of smoking status. The self-reported nature of the data introduces the potential for information bias and misclassification, including social desirability bias, particularly regarding smoking status and NRT use. The absence of multivariable adjustment means that residual confounding by unmeasured variables cannot be excluded. Additional limitations include limited predictor variables in the logistic model, the absence of regional data, and the lack of qualitative methods.

### Future research

Future research should employ probability-based sampling, broader multivariate models, longitudinal designs, confirmatory factor analysis of the adapted NDSS, intervention studies targeting specific subgroups, and mixed-methods approaches to build on the present cross-sectional findings and provide a more comprehensive understanding of NRT awareness and use in Saudi Arabia.

## CONCLUSIONS

The present data demonstrate that while general NRT awareness is high among our convenience sample of Saudi adults, gaps persist in knowledge regarding NRT effectiveness, safety, and appropriate use. The Q-MAT reveals high cessation motivation, while the NDSS indicates a craving-dominant dependence profile. Male gender was the sole significant predictor of NRT awareness. The absence of a significant difference in NDSS scores between motivation groups suggests that motivation level and nicotine dependence severity may not be closely aligned in this sample. Further studies are needed to clarify the implications for cessation support. Longitudinal and interventional studies are warranted to determine whether targeted education, improved access to cessation support, and integration of cessation content into healthcare curricula can effectively address the identified knowledge gaps and contribute to reducing tobacco prevalence in Saudi Arabia.

## Supplementary Material



## Data Availability

The data supporting this research are available from the authors on reasonable request.
