# Supplementary material for: Awareness and perceptions of nicotine replacement therapy among adults in Saudi Arabia: A cross-sectional study
Source: Tob Induc Dis. 2026 Aug 1;24:10.18332/tid/221483. doi: 10.18332/tid/221483 (PMC13450129; doi:10.18332/tid/221483)
Supplement: Supplementary file 1 [file TID-24-135-s1.pdf]

Tobacco Induced Diseases

Supplementary file

© 2026 Almaghlouth F.I. et al.

DOI:
10.18332/tid/221483

The content has been provided by the author(s) and has not been reviewed, verified, or endorsed by European Publishing. It may not have undergone peer review. The views, opinions, and recommendations expressed are solely those of the author(s) and do not necessarily reflect the position of European Publishing. European Publishing accepts no responsibility or liability for any consequences arising from the use of, or reliance on, this content.

SUPPLEMENTARY FILES

Questionnaire on Motivation to Quit (Q-MAT)

Adapted from Aubin H-J, Lagrue G, Legeron P, et al. Questionnaire de motivation à l'arrêt du tabac (Q-MAT). Alcoologie et Addictologie. 2004;26(4):311-316. The Q-MAT composite score (range 0–20) is computed from four scored items (Q1, Q3, Q4, Q6). Items Q2, Q5, and Q7 are descriptive (non-scored) items used for clinical context.

Q1. In six months' time, do you think that: (Scored: 0–8)

- You will still be smoking as much? [Score = 0]
- You will have cut down your cigarette consumption a little? [Score = 2]
- You will have cut down your cigarette consumption a lot? [Score = 4]
- You will have stopped smoking? [Score = 8]

Q2. Do you think that stopping smoking would be good for your self-image? (Descriptive, not scored)

Not at all / A little / A lot / Enormously

Q3. At the moment, do you want to stop smoking? (Scored: 0–3)

- Not at all [Score = 0]
- A little [Score = 1]
- A lot [Score = 2]
- Enormously [Score = 3]

Q4. In the next four weeks, do you think that: (Scored: 0–6)

- You will still be smoking as much? [Score = 0]
- You will have cut down your cigarette consumption a little? [Score = 2]
- You will have cut down your cigarette consumption a lot? [Score = 4]
- You will have stopped smoking? [Score = 6]

Q5. Have you already tried to stop smoking? (Descriptive, not scored)

Never / Once / Several times

Q6. Do you ever feel unhappy about smoking? (Scored: 0–3)

- Never [Score = 0]

• Sometimes [Score = 1]

• Often [Score = 2]

• Very often [Score = 3]

Q7. Do you think that smoking is bad for your health? (Descriptive, not scored)

Not at all / A little / A lot / Enormously

Note: Total Q-MAT score = Q1 + Q3 + Q4 + Q6 (range 0–20). Motivation categories: Low (0–6), Moderate (7–13), High (14–20). Items Q2, Q5, and Q7 are descriptive and are not included in the composite score.

Table 1. *Questionnaire on Motivation to Quit (Q-MAT) Item-Level Response Distribution Among Current Smokers in a Cross-Sectional Online Survey, Saudi Arabia, February–May 2025 (N = 219)*

Q-MAT Item	n	Response	n	%	Score	Scored?
**Q1. 6-month quit intention**	219	Continue same	29	13.24	0	Yes
		Reduce a little	45	20.55	2	
		Reduce a lot	51	23.29	4	
		Quit completely	94	42.92	8	
**Q2. Quitting improves self-image**	220	Not at all	31	14.09	—	No
		A little	46	20.91	—	
		A lot	41	18.64	—	
		Enormously	102	46.36	—	
**Q3. Current desire to quit**	219	Not at all	20	9.13	0	Yes
		A little	55	25.11	1	
		A lot	45	20.55	2	
		Enormously	99	45.21	3	
	219	Continue same	34	15.53	0	Yes

**Q4. 4-week quit intention**		Reduce a little	72	32.88	2	
		Reduce a lot	40	18.26	4	
		Quit completely	73	33.33	6	
**Q5. Previous quit attempts**	219	Never	30	13.70	—	No
		Once	49	22.37	—	
		Several times	140	63.93	—	
**Q6. Dissatisfaction with smoking**	219	Never	14	6.39	0	Yes
		Sometimes	43	19.63	1	
		Often	72	32.88	2	
		Very often	90	41.10	3	
**Q7. Smoking harmful to health**	223	Not at all	6	2.69	—	No
		A little	24	10.76	—	
		A lot	45	20.18	—	
		Enormously	148	66.37	—	

Q-MAT total score (range 0–20) is computed from four scored items (Q1, Q3, Q4, Q6). Items Q2, Q5, and Q7 are descriptive (non-scored) and are not included in the composite score. — = not scored. Q-MAT = Questionnaire on Motivation to Quit.

Table 2. Nicotine Dependence Syndrome Scale (NDSS) Item-Level Response Distribution Among Current Smokers in a Cross-Sectional Online Survey, Saudi Arabia, February–May 2025 (Selected Items)

NDSS Item	n	Not true	Somewhat	Moderate	Very	Extremely
Strong need after abstinence	180	10.56%	15.56%	17.78%	27.78%	28.33%
Regular use throughout day	180	29.44%	22.22%	26.67%	10.56%	11.11%
Need more nicotine (tolerance)	179	24.58%	23.46%	21.79%	13.41%	16.76%
Discomfort after stopping	179	12.85%	21.79%	20.67%	25.70%	18.99%

Emotions influence use	178	11.80%	17.98%	20.79%	20.22%	29.21%
Cannot resist cravings	179	16.20%	22.91%	26.82%	19.55%	14.53%
Craving after few hours	183	10.93%	22.40%	19.13%	24.04%	23.50%

Scale: 1 = Not at all true to 5 = Extremely true. n varies across items due to item-level non-response. NDSS = Nicotine Dependence Syndrome Scale.
